# Spatial price differences of medical services: evidence from the Yangtze River Delta in China

**DOI:** 10.1186/s12913-023-09774-0

**Published:** 2023-07-17

**Authors:** Luo Li, Bao Liu

**Affiliations:** 1 Department of Health Economics, School of Public Health, Fudan University, Shanghai, China; 2 Key Laboratory of Health Technology Assessment (Fudan University), Ministry of Health, Shanghai, China

**Keywords:** Medical services, Spatial price index, Price differences, The Yangtze River Delta

## Abstract

**Background:**

Price differences of medical services across regions may affect equity in health financing. This study aimed to estimate the spatial price index of medical services to measure price levels across regions in the Yangtze River Delta, China.

**Methods:**

Gini-Éltetö-Köves-Szulc method and minimum spanning tree method based on the purchasing power parities were used in this study.

**Results:**

According to the Gini-Éltetö-Köves-Szulc method, Shanghai and Anhui province had price levels that are 127.55% and 103.45% respectively of the price level in Zhejiang province, whereas in Jiangsu medical services were priced at 92.71% of that in Zhejiang province. The spatial price index of medical services in the Yangtze River Delta based on the minimum spanning tree method provided similar results.

**Conclusions:**

Regions in the Yangtze River Delta had significant gaps in medical services price levels. And the price levels tended to not correlate with socioeconomic levels. It is necessary to promote the regional coordination of medical services price and better achieve equity in health.

## Background

Differences in price levels across regions can reflect many factors such as transportation costs, and it should be taken into consideration when measuring economic activities such as gross domestic product (GDP) and income levels [1]. For geographically large countries, comparing prices across regions is crucial for evaluating and ensuring balanced development across regions within the country [2]. Therefore, both government officials and researchers are interested in estimating spatial price indexes to measure regional price differences for one period [3]. In the U.S., the Bureau of Economic Analysis (BEA) has officially published annual regional price parities (RPPs) that measure price level differences across states and metropolitan areas [4]. With the development of the European integration, the importance of regional price levels has become more and more obvious. For example, the GDP per capita adjusted for regional price levels may vary from the non-adjusted, which could lead to a different classification of regions into groups eligible for funding under the European Union (EU) regional policy [5]. Additionally, there have been numerous studies on price comparisons across regions in the healthcare sector, with a focus on drugs and consumables prices [6–8].

In this context, a measure of the price paid for medical services across regions is of equal importance. Medical services price reflects the value of physician services. It ensures that the costs of delivering services are covered, and provides incentives for health care providers [9]. Medical services price is also a determinant of health expenditures. Related studies have shown that the price factor can account for almost 50% of the growth in health care expenditures per capita in the U.S [10]. With the rising concern of health spending, policy-makers have increasingly focused on medical services price. Medical services price levels may vary across regions due to different cost levels, and regional price differences account for half of the variation in health spending for the privately insured [11]. As a result, it is of great importance to measure the price of medical services across regions. It can help explain why health spending varies across regions, and thus help adjust future health expenditure decisions and improve efficiency [12]. What’s more, price levels may affect people’s burden of out-of-pocket payment. The significant regional price differences for medical services may impact the equal access for those living in areas with higher prices, especially for low-income populations, to medical services. Therefore, it is important to study on spatial price differences to identify the current status of regional price levels of medical services. Some countries have considered the price differences of medical services across regions. For example, the Centers for Medicare & Medicaid Services in the U.S. has used Geographic Practice Cost Index to adjust fee-for-service payments in the Medicare Physician Fee Schedule and therefore account for differences in costs due to geographic location [13]. NHS England has calculated the market forces factor presented in the payment index to vary the prices to reflect differences in unavoidable costs between providers [14]. The Eurostat and the OECD have also calculated price level indices of health and hospital to compare price levels across regions [15].

China has continued to strengthen the central role in the management of medical services price since the 21st century and introduced a national specification of medical services price schedule in 2001. It should be emphasized that only physician services, not other elements like pharmaceuticals, are included in medical services in China. Based on the national specification, the price administration of each province in China has established its own medical services price schedule and set the price limits for public medical institutions including tertiary hospitals, secondary hospitals, and community health service centers within the province, which has also resulted in price differences of medical services across regions. In some provinces, the price management of certain medical services is decentralized to the prefecture-level, enabling them to set price without exceeding the provincial price. The price limit set by the government was to emphasize the commonweal of public medical institutions and ensure the equal access to medical services. However, such price may not fully cover the cost of medical services. Therefore, in recent years, China has implemented medical services price reform and gradually established the dynamic adjustment mechanism for medical services price based on the change of cost and hospital revenue structure. In this context, the study of regional price differences can also provide useful references for adjusting and establishing price levels in different regions. What’s more, China has introduced a coordinated regional development strategy in recent years, especially with the integrated development of the Yangtze River Delta region becoming a national strategy. Similar to the EU region, regions in the Yangtze River Delta have reaped major benefits from its integrated development during the past years, not only in terms of high-quality economic development but also in the field of healthcare. The public services including medical services are delivered in a more equitable way, as the imbalance of healthcare resource supply including human, material, and financial resources in the Yangtze River Delta has significantly decreased [16]. And trans-regional medical institutions have been tried in the Yangtze River Delta region to optimize the allocation of medical resources. This also draws our attention to the price levels of medical services across regions in the Yangtze River Delta. At the same time, while the development of regional coordination is beneficial to trans-provincial medical treatment, it also raises great concerns because the medical insurance funds have to face the price levels of other regions. If regional medical services price differs too much, regions with smaller medical insurance fund balances would be even less able to cover the medical expenses incurred by too many patients seeking treatment in higher-price regions, which would be detrimental to sustainability of medical insurance funds. Additionally, China started paying more attention to the coordination between medical services price and medical insurance payments since the establishment of the National Healthcare Security Administration in 2018. As a result, the National Healthcare Security Administration of China has been more concerned about the price differences across provincial regions and proposed that medical services price in neighboring provinces should keep reasonable differences, as the medical insurance funds shall be progressively put under social pooling at provincial level.

Considering the context mentioned above and the disparities of medical resources in China [17], close attention should be paid to the differences in the price levels of medical services across regions. From the literature review, medical services price comparisons were often made by time, which could reflect the price change over time [18]. No study, however, has estimated the spatial price index of medical services and measured the price differences across regions in the Yangtze River Delta, China. Taking these needs into account, the present study aimed to estimate the spatial price index of medical services to measure price levels across regions in China. However, it is important to note that our study only focused on the price levels of medical services in public medical institutions. Firstly, the price limits in China only apply to public medical institutions, while the price of non-public medical institutions is fully market-driven. Therefore, studying the price levels of public medical institutions has more policy implications. Secondly, the majority of medical services in China are delivered by public medical institutions, which limits the impact of non-public medical institutions’ price levels. What’s more, it is also very difficult to collect relevant data on the price of non-public medical institutions. The provincial regions in the Yangtze River Delta present an ideal case study. Although the management of medical services price is decentralized at the province level in China [19], the four provincial regions in the Yangtze River Delta followed as Shanghai, Jiangsu province, Zhejiang province, and Anhui province all have similar medical services price schedule to make the comparison possible and ensure comparability. What’s more, after the implementation of the Zero-Markup Drug Policy in 2015 [20], medical services price in the Yangtze River Delta region has been adjusted for several times. It’s still unclear the current price levels across regions.

The remainder of the paper is organized as follows: Sect. 2 deals with data and methodology. The estimate of the spatial price index of medical services in the Yangtze River Delta region is presented in Sect. 3, while the remaining section concludes and discusses questions for further research.

## Methods

### Data and statistics

**Fig. 1:**
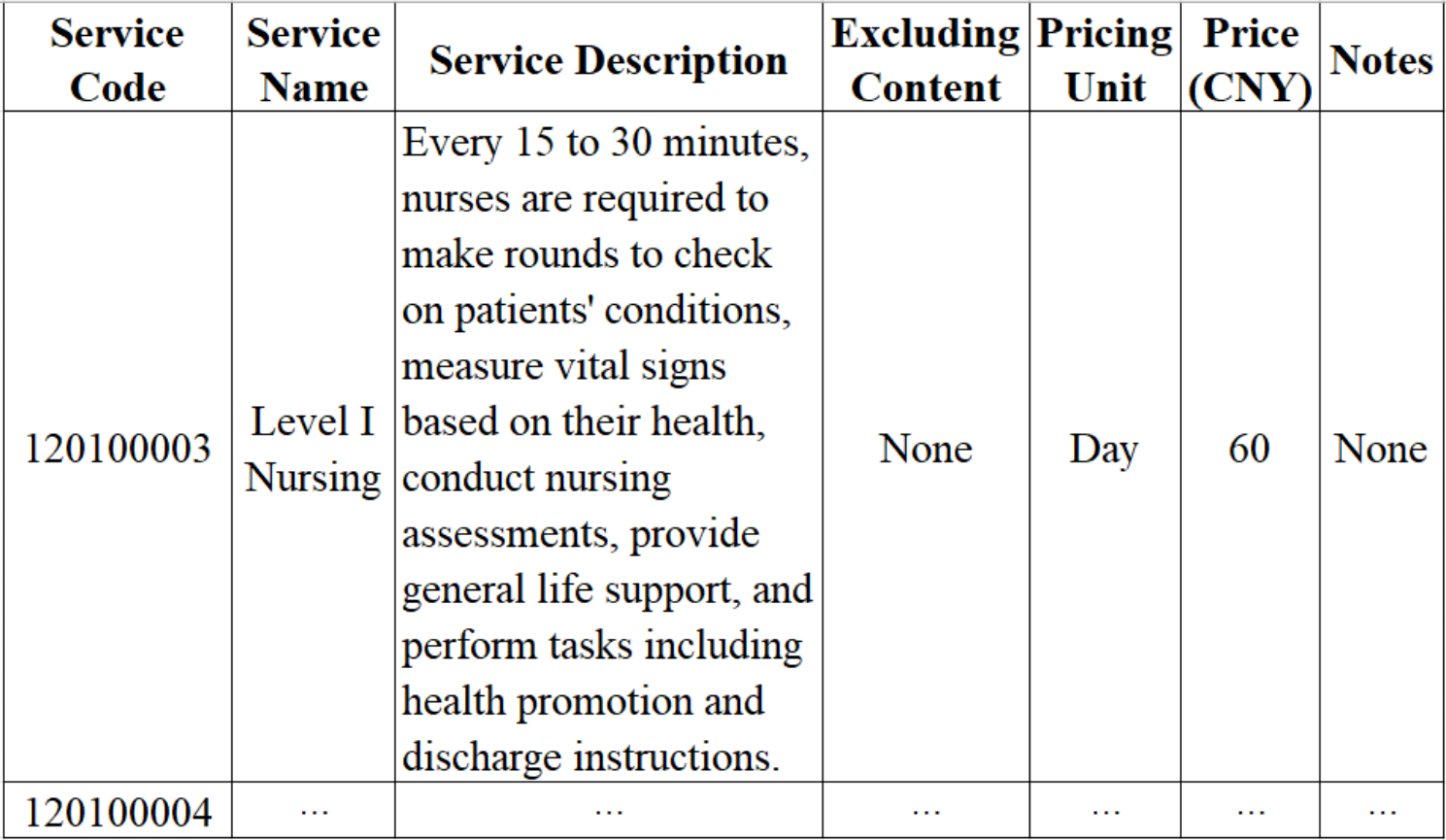
The template of medical services price schedule in each region. Note: This was selected from the price schedule of Shanghai and translated into English. The price schedules of the other three provinces in the Yangtze River Delta were similar to this

Medical services price schedules were collected from the official websites of the Health Commission and the Healthcare Security Administration of each region. And the price schedules were updated up to December 2020 based on relevant price adjustment policies. As shown in Fig. 1, the price schedule in each region included service code, service name, service price, etc. The highest prices in each region were selected in this study to guarantee comparability. In order to further ensure comparability, the features of medical service, such as codes, names, etc., were used to match the same service across four regions.

The expenditure weights of medical services were also needed to estimate the spatial price index, which meant the amounts of medical services being consumed. Real world data and literature review were used to calculate weights for different categories of medical services in each region. More specifically, the 2019 data of medical services charge for all public medical institutions in Shanghai collected from the Shanghai Health Information Center, with a total of 2.65 billion records, were used to calculate the weights of Shanghai. We were able to identify the corresponding category to which the service belonged because each record contained the code of the medical service. This allowed us to aggregate and calculate the total amount for each category and the corresponding expenditure weights. The expenditure weights of Jiangsu province were computed using data collected from a hospital revenue structure survey conducted by the Healthcare Security Administration of Jiangsu Province in 2018. The survey included 311 medical institutions in Jiangsu province and provided data on amounts for different categories of medical services. We were able to calculate the total amount and corresponding expenditure weights for each category using this data. Data for Shanghai and Jiangsu in the year 2020 was not available to us. However, considering that there were no significant changes in disease spectrum, clinical pathways, or price adjustments from 2018 to 2020 in these regions, we believe that the expenditure weights derived above can reflect the situation in 2020. The calculation of weights in Anhui and Zhejiang was similar to that in Jiangsu, the difference was that we cannot directly obtain the amount data for different categories of medical services. Instead, we collected these data through literature [21, 22].

The Consumer Price Index (CPI) for medical services of each region were used to estimate medical services price levels from 2015 to 2019 for time-series comparisons. R4.0.2 software was used for descriptive statistical analysis and spatial price index estimation.

### Spatial price index estimation

When measuring price differences of medical services across regions, more than two regions would be involved in the comparison. So, the spatial price index is constructed as a multilateral price index rather than a bilateral price index for comparison between two regions. Compared with the bilateral price index, the multilateral price index is required to satisfy several fundamental properties such as transitivity [2, 23], which means that the direct price index between any two regions yields the same result as an indirect comparison via any other region [24]. Purchasing power parities (PPPs) has been a widely used multilateral index for comparing overall price levels and specific categories such as food and healthcare price [25], which showed the ratio of prices for the same basket of goods and services in different regions and was developed by the International Comparison Program (ICP) [24, 26]. This study used PPPs to estimate the spatial price index of medical services to measure price levels across regions in the Yangtze River Delta. The detailed steps are as follows.

#### The basic heading index

In the first step, it was necessary to define the basket of medical services and divide these services into several basic headings, which was defined as a group of similar services. The same services across four regions were included as the basket. As mentioned above, the price schedule in each region was based on the national specification and medical services were all divided into four categories based on service’s property followed as general medical services, medical diagnosis services, clinical treatment services, and traditional medicine services. The four categories served as the basic heading. Then the basic heading index between every two regions could be estimated using some methods. One method was the Jevons index, which was the geometric mean of the price ratio of each service in the basic heading between two regions. Another one was the Country Product Dummy (CPD) method, which used price data to build a regression model to estimate the basic heading index. If every province set a price for every medical service, which was the case in this study, the results from the two methods would be equivalent [24]. Therefore, Jevons index was used to estimate the basic heading index. The formula of Jevons index is shown as follows:

1 $${PPP}_{jk}^{Jevons}=\prod _{i}^{N}{\left[\frac{{P}_{ik}}{{P}_{ij}}\right]}^{1/N}$$

where ${P}_{ik}$ represents the price of *i-th* medical service in region *k* and ${P}_{ij}$ represents the price of *i-th* medical service in region *j* (*i* = 1, 2, …, N, N is the total number of medical services in the basket).

#### Aggregation above basic headings

In the second step, the expenditure weights data in each region were used to aggregate basic heading indexes into a bilateral price index between every two regions. There were also different types of the bilateral index, including the Laspeyres index, the Paasche index, and the Fisher index. The Fisher index was the geometric mean of Laspeyres index and Paasche index, and it was used to aggregate the results from basic heading. These indexes were calculated using the following formulas:

2 $${PPP}_{jk}^{\text{L}\text{a}\text{s}\text{p}\text{e}\text{y}\text{r}\text{e}\text{s}}=\frac{\sum _{i}^{N}{p}_{ik}\bullet {q}_{ij}}{\sum _{i}^{N}{p}_{ij}\bullet {q}_{ij}}=\sum _{i}^{N}{PPP}_{i}^{jk}\bullet {W}_{ij}$$

3 $${PPP}_{jk}^{\text{P}\text{a}\text{a}\text{s}\text{c}\text{h}\text{e}}=\frac{\sum _{i}^{N}{p}_{ik}\bullet {q}_{ik}}{\sum _{i}^{N}{p}_{ij}\bullet {q}_{ik}}=\frac{1}{\sum _{i}^{N}\frac{1}{{PPP}_{i}^{jk}}\bullet {W}_{ik}}$$

4 $${PPP}_{jk}^{Fisher}={\left({PPP}_{jk}^{\text{L}\text{a}\text{s}\text{p}\text{e}\text{y}\text{r}\text{e}\text{s}}\bullet {PPP}_{jk}^{\text{P}\text{a}\text{a}\text{s}\text{c}\text{h}\text{e}}\right)}^{1/2}$$

where ${W}_{ij}$ represents the expenditure weight of *i-th* basic heading in region *j* and ${W}_{ik}$ represents the expenditure weight of *i-th* basic heading in region *k* (*i* = 1, 2, …, N).

#### The multilateral index

In the final stage, the bilateral index between every two regions was adjusted to the multilateral index which could measure price differences across regions. With the development of ICP, different methods have been proposed to calculate the multilateral index. This study used two methods to estimate the spatial price index of medical services. The first one was the Gini-Éltetö-Köves-Szulc (GEKS) method, which originated with Gini (1930), and was independently rediscovered by Éltetö and Köves (1964) and Szulc (1964). It’s the standard method mainly used in ICP, which has the advantage of that each region is treated in a symmetric way and is fully consistent with the economic approach to index number theory [27]. Let ${F}_{ji}$ represents the Fisher index, and the multilateral index of region *j* and *k* using GEKS method can be given by:

5 $${GEKS}_{jk}=\prod\limits_{i=1}^{M}{\left[{F}_{ji}\bullet {F}_{ik}\right]}^{1/M}$$

Where ${F}_{ji}$ represents the Fisher index between region *j* and *i*, ${F}_{ik}$ represents the Fisher index between region *i* and *k*, and M represents the number of regions for comparison.

Another method was the minimum spanning tree (MST) method, which was introduced by R. J. Hill (1999). It’s an alternative to the GEKS method and could be considered for use in future ICP round, which has the advantages that it uses a superlative index number formula for forming bilateral links and takes into account substitution effects [27]. The multilateral index based on MST method was calculated through a spanning tree from the *Paasche-Laspeyres Spread*, which showed the similarity between the Paasche index and the Laspeyres index [28] and was given by:

6 $${PLS}_{jk}=\left|log\frac{{PPP}_{jk}^{\text{L}\text{a}\text{s}\text{p}\text{e}\text{y}\text{r}\text{e}\text{s}}}{{PPP}_{jk}^{\text{P}\text{a}\text{a}\text{s}\text{c}\text{h}\text{e}}}\right|$$

## Results

### Descriptive statistical analyses

There were more than 4000 different medical services listed on the price schedule for each region in the Yangtze River Delta. As shown in Table 1, the average price of medical services in Shanghai topped the rest by 1 041.59 yuan, while Jiangsu Province was the lowest at 768.57 yuan. After comparability matching, there were a total of 3025 medical services items left.

**Table 1:** Results of descriptive statistics on medical services price of each region (CNY).

Region	Mean	Max	Min	Median (Q_1_, Q_3_)
Shanghai	1 041.59	27 000.00	1.00	200.00 (30.00,1 600.00)
Jiangsu	768.57	31 500.00	0.10	130.00 (26.00,1 200.00)
Zhejiang	997.85	39 600.00	0.00	172.50 (23.00,1 476.00)
Anhui	785.73	18 750.00	0.00	144.00 (25.00,1 125.00)

### Results of spatial price index

Table 2 shows the results of Fisher index between every two regions in the Yangtze River Delta. The region listed in each column is the reference region. For example, the number in the second row and first column is 73.22, indicating that the Fisher index of medical services in Jiangsu province is 73.22 when compared to Shanghai (100). The following tables are all displayed using the same rule.

**Table 2:** Fisher index between every two regions in the Yangtze River Delta

Region	Shanghai	Jiangsu	Zhejiang	Anhui
Shanghai	100	136.58	128.11	123.66
Jiangsu	73.22	100	93.43	88.27
Zhejiang	78.06	107.03	100	97.85
Anhui	80.86	113.29	102.20	100

GEKS method was firstly used to estimate the spatial price index. As shown in Table 3, when Zhejiang province was set as the reference region, Shanghai and Anhui province had price levels that are 127.55% and 103.45% respectively of the price level in Zhejiang province, whereas in Jiangsu province medical services were priced at 92.71% of that in Zhejiang province. The spatial price index of medical services in the Yangtze River Delta based on the MST method provided similar results (Table 4).

**Table 3:** Spatial price index of medical services based on GEKS method

Region	Shanghai	Jiangsu	Zhejiang	Anhui
Shanghai	100	137.59	127.55	123.30
Jiangsu	72.68	100	92.71	89.62
Zhejiang	78.40	107.87	100	96.67
Anhui	81.10	111.59	103.45	100

**Fig. 2:**
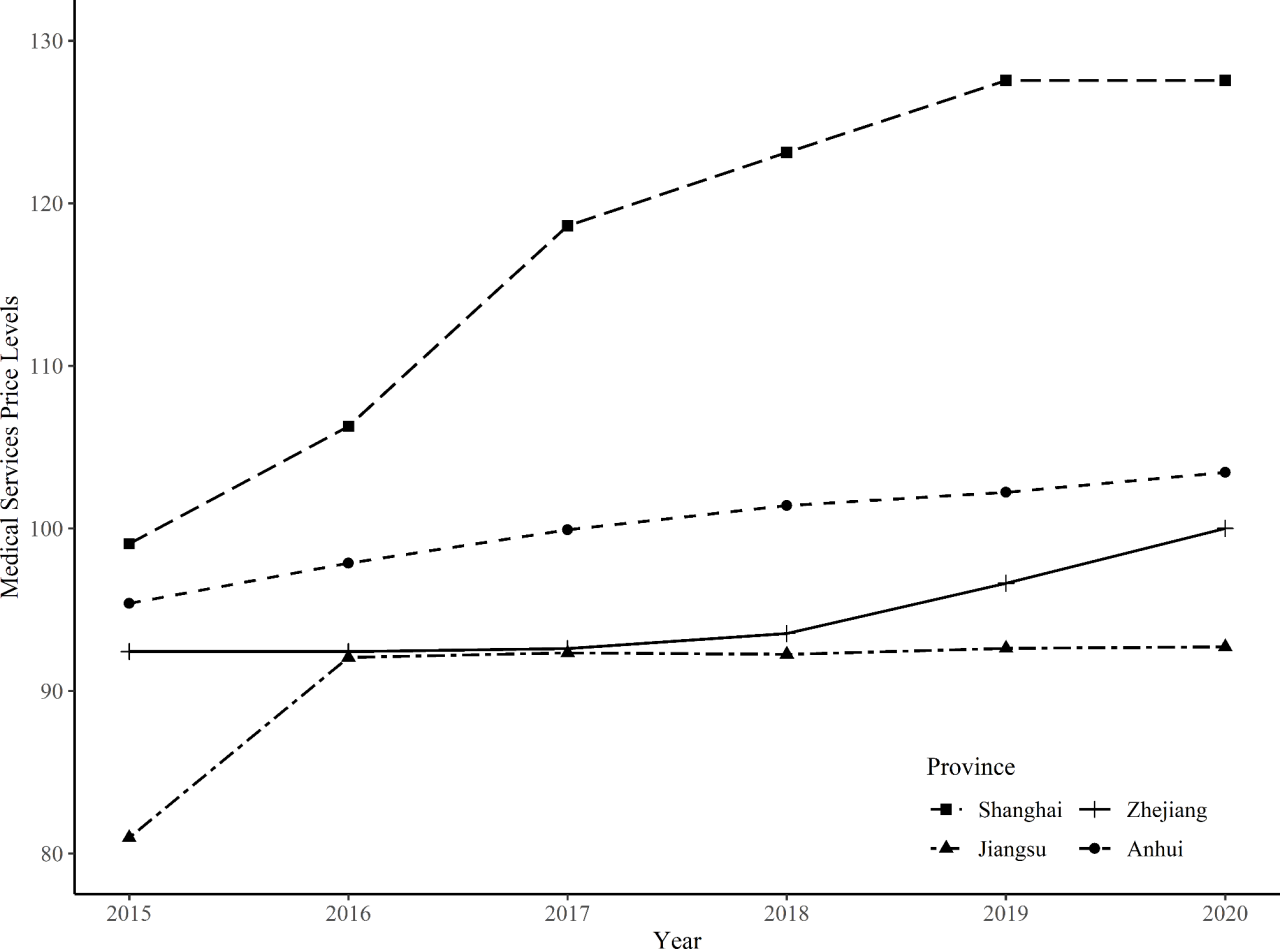
Changes in the price levels of medical services from 2015 to 2020. Note: Zhejiang province in 2020 = 100, based on GEKS methods

The price changes of medical services in each region from 2015 to 2020 were shown in Fig. 2. The price levels of medical services have been increasing in all four regions, with Shanghai remaining at the highest level and Jiangsu remaining at the lowest. In 2015, the price levels of medical services in Shanghai and Anhui province were 107.17% and 103.20% of those in Zhejiang province, respectively, whereas that in Jiangsu province was 87.61%.

**Table 4:** Spatial price index of medical services based on MST method

Region	Shanghai	Jiangsu	Zhejiang	Anhui
Shanghai	100	136.58	128.11	125.36
Jiangsu	73.22	100	93.80	91.79
Zhejiang	78.06	106.61	100	97.85
Anhui	79.78	108.96	102.20	100

## Discussion

To our best knowledge, this study is the first to estimate the spatial price index to compare price levels of medical services in the Yangtze River Delta, China. According to the results, this study found that the regions in the Yangtze River Delta had significant gaps in medical services price levels. Among the regions in the Yangtze River Delta, Shanghai has the highest price level of medical services, followed by Anhui, Zhejiang, and Jiangsu Provinces. And the price differences across regions have been larger in 2020 compared to 2015. It’s of great practical importance to estimate the spatial price index of medical services. The Yangtze River Delta has become the region with the largest scale of trans-provincial medical treatment in China [29], which is the challenge brought about by the integrated development. Therefore, the regions in the Yangtze River Delta should pay attention to controlling the differences in the price levels of medical services so as to better achieve equity in health financing. And when comparing health expenditure across regions, price levels should also be taken into account. With the advancement of medical services price reform in China, the spatial price index of medical services could be used as a decision tool to examine the effects of price changes on regional price levels and to provide scientific references for future price adjustments.

**Fig. 3:**
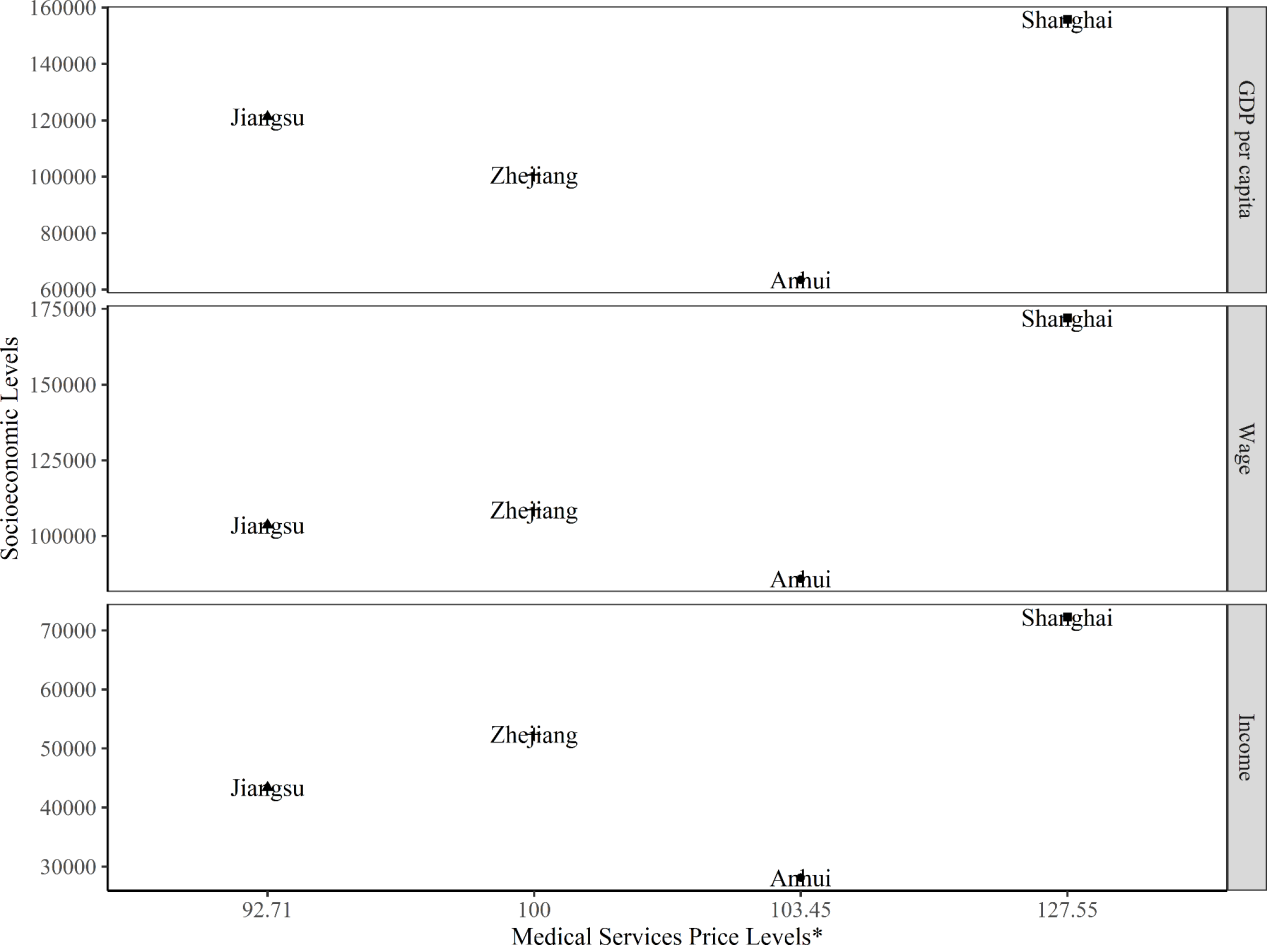
Comparison of medical services price levels and socioeconomic levels. Note: *: Zhejiang province = 100, based on GEKS methods; GDP per capita: Gross Domestic Product per capita, CNY, 2020; Wage: Average Wage of Employed Persons in Urban, CNY, 2020; Income: Disposable Income Per Capita, CNY, 2020; Source: The National Bureau of Statistics of China

Previous studies have found that price levels across regions should be positively correlated with socioeconomic status such as income levels [1, 30, 31]. This is not consistent with the findings of our study. As shown in Fig. 3, Shanghai has the greatest level in terms of economic and social development indicators such as GDP per capita, average wage, and disposable income, whereas Anhui province has the lowest. But the price level of medical services in Anhui province ranked second compared with other regions in the Yangtze River Delta. The price levels of medical services across regions in the Yangtze River Delta tended to not correlate with socioeconomic levels. Medical services price in China is established by the government in the interest of the public welfare, which is different from products and services in a conventional market. This may help explain this finding. As mentioned above, medical services price in the Yangtze River Delta region has been adjusted for several times. Regions in the Yangtze River Delta may also have given less consideration to regional price levels when setting and adjusting medical services prices, which may also lead to an increasing price difference. This finding also indicates that regions in the Yangtze River Delta should promote the regional coordinated development of medical services price and determine the acceptable range of price differences across regions.

This study conducted a cross-sectional analysis of medical services price levels in the Yangtze River Delta. As medical services are often supplied by governments, previous studies have used “quasi-prices”, which is negotiated between payers and providers, to compare price across regions [30]. Instead of only physician services, the quasi-prices might include medical costs such as pharmaceuticals and medical goods. This study used the exact price data of medical services so that the results can reflect the price levels across regions directly. And this study used two different methods to estimate the spatial price index to check the robustness of results. The comparability is an important principle in the regional price comparison. Differences in goods or services quality could lead to the underestimation or overestimation of price levels [24]. This study matched the same services using service codes, names, etc., to obtain comparable medical service items among the regions in the Yangtze River Delta. However, it should be noted that the quality of medical services depends on service process and patients and provider interactions, making it difficult to measure and account for differences in the quality of medical services [32]. Therefore, the methodology which could adequately account for quality differences needs to be further explored, so that a more accurate price index could be constructed to scientifically reflect the price levels across regions. In addition, this study obtained price levels of medical services in other years using CPI, which was merely a preliminary estimate. Once the systematic methodology for estimating the spatial price index of medical services has been established, future studies will be possible to continuously track and measure the changes in medical services price levels across regions more accurately and constitute panel data, thus providing more information on price differences across regions.

This study also has the following limitations. Firstly, we only calculated the price levels for four provincial regions due to data availability, so we only directly compared price levels with the socioeconomic level instead of conducting a more rigorous regression analysis. Therefore, our future work will focus on addressing this issue, which will enable us to include a larger sample size to measure price levels and further explore the factors account for the price differences using fuzzy set Qualitative Comparative Analysis [33]. Secondly, this study used the highest prices in each region, but the medical services price may vary within regions due to the decentralization. So, this may affect the representativeness of research data. What’s more, this study used expenditure weights data collected from the literature review due to the data availability. It might be different from the actual data, which could have an impact on the outcomes. This also points to the need for improved data infrastructure to facilitate the estimation of spatial price index.

## Conclusions

Regions in the Yangtze River Delta had significant gaps in medical services price levels. And the price levels of medical services across regions in the Yangtze River Delta tended to not correlate with socioeconomic levels. With the development of regional coordination, this will raise concerns about the sustainability of medical insurance funds and the equity in health financing. Therefore, provinces and cities in the Yangtze River Delta region, as well as other regions, should focus on the price differences of medical services across regions. It is necessary to further optimize the price levels, improve the methodological system of medical services price comparison, and promote the integration of medical insurance to better achieve equity in health.

## Data Availability

The price schedules data of regions in the Yangtze River Delta and the expenditure weights data of Zhejiang and Anhui are available from the corresponding author on reasonable request. The expenditure weights data of Shanghai and Jiangsu are not publicly available due to the data ownership belongs to the governments of regions in the Yangtze River Delta.

## List of Abbreviations

GDP: Gross domestic product
BEA: The Bureau of Economic Analysis
EU: European Union
RPPs: Regional price parities
PPPs: Purchasing power parities
ICP: International Comparison Program
CPD: Country Product Dummy
GEKS: Gini-Éltetö-Köves-Szulc
MST: Minimum spanning tree
CPI: Consumer Price Index
